# RB116: An RB1+ retinoblastoma cell line expressing primitive markers

**Published:** 2012-11-29

**Authors:** Anthony Bejjani, Mee Rim Choi, Linda Cassidy, David W. Collins, Joan M. O’Brien, Tim Murray, Bruce R. Ksander, Gail M. Seigel

**Affiliations:** 1 University at Buffalo School of Medicine and Biomedical Sciences, Buffalo, NY; 2 University at Buffalo, Center for Hearing & Deafness, SUNY Eye Institute, Buffalo, NY; 3 Scheie Eye Institute, University of Pennsylvania, Philadelphia, PA; 4 Bascom Palmer Eye Institute, University of Miami, Miami, FL; 5 Schepens Eye Research Institute, Massachusetts Eye and Ear

## Abstract

**Purpose:**

Retinoblastoma (RB), an intraocular tumor of childhood, is commonly associated with mutations in the *RB1* gene. RB116 is a novel, early passage RB cell line that has not been previously characterized. In this study, we examined RB116 for the expression of RB1 and tested the hypothesis that RB116 cells would express stem cell markers as well as retinal progenitor cell markers. We compared RB116 cells with other well known RB cell lines, including Y79 and WERI-RB27.

**Methods:**

We evaluated expression of *RB1* in RB116 cells by sequencing, multiplex ligation-dependent probe amplification, quantitative reverse transcriptase polymerase chain reaction (qRT–PCR), western immunoblot, and immunocytochemistry. Next, RB116 cells, along with Y79 and WERI-RB27 cells, were examined for expression of stem cell markers (ABCG2, Nanog, Oct3/4, ALDH1A1) and retinal progenitor markers (PAX6, CHX10) by quantitative immunocytochemistry. Immunocytochemical findings were accompanied by PCR analysis.

**Results:**

RB116 cells expressed *RB1* at the mRNA and protein levels, with no mutations detected by either sequencing analysis, or gene dosage abnormalities detected by multiplex ligation-dependent probe amplification. The RB1 protein was immunoreactive in RB116 cells with an atypical perinuclear localization. RB116 cells also expressed stem cell markers, with 3%–5% of cells immunopositive for ABCG2, Oct3/4 and ALDH1A1, with at least 18% of cells immunoreactive to Nanog. These findings were confirmed by RT–PCR. Small percentages of RB116 cells also exhibited immunoreactivity to retinal progenitor markers PAX6 (9.8%) and CHX10 (1.2%). Expression of mRNAs for these markers was confirmed by qRT-PCR.

**Conclusions:**

RB116 cells demonstrate RB1 expression accompanied by atypical perinuclear localization. RB116 cells also express primitive stem cell and retinal progenitor cell markers. Further studies on the phenotypes of both RB1-positive and RB1-negative human RB cells may be important in assessing differentiation potential of these cells, as well as designing targeted differentiation therapies.

## Introduction

Retinoblastoma (RB) is an intraocular tumor that most commonly manifests in early childhood. RB was one of the earliest childhood tumors [1] to be characterized at the molecular level [2,3], with the discovery of the *RB1* tumor susceptibility gene on chromosome 13 [4] that exhibits tumor suppressor properties [5]. Loss of RB1 function is associated with a variety of human cancers, while inactivation of the *RB1* tumor suppressor gene has been reported in several human malignancies in addition to RB [6], such as cancers of the breast [7,8], prostate [9], and lung [10]. Furthermore, the *RB1* gene family is intimately involved in the control of cellular proliferation, survival, and differentiation pathways in many mammalian cells [11].

RB116 is a low passage cell line established from an RB tumor that has not been well characterized. In contrast, RB cell lines such as Y79 [12] and WERI-RB27 [13] are well characterized genetically [14] with known *RB1* mutations [13,15], and have been in culture for many years. Our group has identified stem cell marker expression in both Y79 and WERI-RB27 cells [16,17], a finding supported by additional studies of clinical RB samples [18–20]. However, it is unclear whether an early passage cell line, such as RB116 would retain these stem cell markers. In this study, we have identified RB116 as an RB1-expressing cell line that contains subpopulations of cells that express markers consistent with stem cells and retinal progenitor cells.

## Methods

### Cell culture

The RB116 cell line was initially established from an explant of a large primary human RB tumor and provided for this study without patient identifiers. Human subject protections were maintained according to the Declaration of Helsinki and approved Institutional Review Board (IRB) protocols. RB cell lines Y79, WERI-RB27, and RB116 were grown in suspension under standard culture conditions (37 °C with 95% air, 5% CO_2_). RB116 cells were grown in RPMI medium with 1% 4-(2-hydroxyethyl)-1-piperazineethanesulfonic acid (HEPES; Life Technologies, Carlsbad, CA) and 10% calf serum (Gibco, Grand Island, NY). Y79 and WERI-RB27 cells were grown in Dulbecco's Modified Eagle's Medium (DMEM; Sigma, St. Louis, MO) with 10% calf serum. MDA-MB231 (HTB-26, American Type Culture Collection, Manassas, VA), a human breast adenocarcinoma cell line, was grown as a control RB1-expressing cancer cell line in Liebovitz’s L-15 medium with 10% fetal bovine serum at 37 °C in 100% room air.

### Polymerase chain reaction amplification of *RB1* exons

Genomic DNA from RB116 cells was isolated using a Qiagen DNA mini kit (# 51,104). *RB1*’s 27 exons were amplified using 25 PCR reactions run in parallel under the same cycling conditions; exons 15,16 and exons 22,23 are closely linked, and each pair was contained within a single amplicon (primer sequences available upon request). PCR reactions contained 20 ng of genomic DNA, 4 pmol of forward and reverse primers, 0.5 units of Invitrogen Platinum Taq (Invitrogen, Carlsbad, CA, 10,966–034) 2.5 mM MgCl_2_, 1.5 M betaine, and 1x PlatinumTaq PCR buffer in a total volume of 10 μl. Thermal cycling was done using the following touchdown protocol: 95 °C for 5 m; 14 cycles (94 °C for 20 s, 63.0–56.0 °C for 20 s with 0.5 °C step down per cycle, 72 °C for 45 s); 25 cycles (94 °C for 20 s, 56 °C for 20 s, 72 °C for 45 s); 72 °C for 10 min; hold at 4 °C. PCR reactions were cleaned by up SAP/Exo1 digestion: 1 unit of shrimp alkaline phosphatase (USB cat. # 70092Y) and 1 unit of exonuclease 1 (USB cat. # 70073Z; USB Corporation, Cleveland, OH) in 4 μl was added to each 10 μl PCR reaction. Samples were incubated for 1 h at 37 °C, followed by 15 min at 95 degrees to inactivate enzymes.

### Capillary electrophoresis Sanger sequencing

Cycle sequencing was done with the Applied Biosystems BigDye Terminator 3.1 kit (#4336919; Life Technologies, Carlsbad, CA) using 6 pmol of forward or reverse sequencing primer, and 1.25 μl of the digested PCR product in a volume of 5 μl, and cycled as follows: 96 °C for 1 m; 25 cycles (96 °C for 10 s, 50 °C for 5 s, 60 °C for 4 min); hold at 4 °C. Sequencing reactions were cleaned up with BigDye XTerminator purification kits (Applied Biosystems #4376487) and resolved by capillary electrophoresis using an Applied Biosystems 3730 genetic analyzer and POP-7 polymer. Sequencing Analysis 5.2 software (Applied Biosystems) was used for basecalling with default settings, and Sequencher v4.1 software (Gene Codes Corp.) was used to align sequencing chromatograms to the *RB1* reference sequence (NG_009009.1) and screen all exons, splice junctions, and the proximal 5′ promoter region for pathogenic variants.

### Multiplex ligation-dependent probe amplification

Multiplex ligation-dependent probe amplification (MLPA) gene dosage assay was performed with reagents from MRC Holland (kit #P047 with FAM labeled primers), targeting 23 of *RB1*’s 27 exons and 3 nearby genes (*DLEU1*, *CHC1L*, *ITM2B*). No probes targeted exons 5, 10, 15, or 16 of *RB1*. MLPA reaction products were resolved on a 3130×l genetic analyzer with 50 cm capillary array and quantified with GeneMarker v1.7 (Softgenetics, State College, PA) fragment analysis software using default settings and analysis by the MLPA ratio method and quantification by peak height. Peak heights were normalized using a synthetic control derived from two normal human blood specimens.

### Sodium dodecyl sulfate PAGE and western blotting

RB116, Y79, and U87-MG glioma cells were pelleted and lysed in 2x Laemmli buffer (125 mM Tris-Hcl), 10% glycerol, 10% sodium dodecyl sulfate) with 1% Sigma protease inhibitor cocktail (#P8340; Sigma-Aldrich, St. Louis, MO), boiled for 10 min, centrifuged 10 min, at 14,000 g. Protein concentrations were determined with the Bio-Rad (Hercules, CA) DC assay (#500–001) and proteins separated by sodium dodecyl sulfate PAGE using a 4%–12% Bis-Tris Novex NuPage gel (Invitrogen # NP0322BOX; Life Technologies) run for 90 min at 200 V, with 20 μg total protein loaded per lane, versus MW size standard (Amersham #RPN-800; GE Healthcare Lifesciences, Pittsburgh, PA). Two nanograms of recombinant purified RB1 protein were used as a positive control, alongside 20 μg MOLT-4 cell extract. The gel was transferred to a polyvinylidene difluoride(PVDF) membrane in 3-(N-morpholino)propanesulfonic acid (MOPS) buffer with 10% methanol at 36V for 65 min. The blots were blocked with 5% nonfat milk in Tris-buffered saline–Tween at 4 °C, then incubated with RB1 monoclonal antibody QED 3107 (targeting aa 886–905, diluted 1:5000) or BD 554,136 (targeting aa 332–344, diluted 1:5000) for 4 h at room temperature (RT). Blots were incubated for 1 h with mouse Immunoglobulin G (IgG)–horseradish peroxidase–conjugated secondary antibody (Santa Cruz SC-2005) diluted 1:20,000 and bands detected by chemiluminescence using the Amersham ECL Advance kit (#RPN2135) with Hyperfilm ECL. A blot was stripped and reprobed with alpha-tubulin antibody (Santa Cruz SC-8035), diluted 1:1000, to evaluate protein loading.

### Immunocytochemistry

Immunocytochemistry was performed as per our previous studies [16,17]. Briefly, cells were grown, centrifuged, rinsed, fixed in cytospin solution (72% isopropyl alcohol, 19% acetone, 7.6% glycerol) and spun onto slides with a Shandon Cytospin II. Cytospins were blocked with enzyme/protein block (Abcam, Cambridge, MA) and then incubated in primary antibody (Table 1) or isotype control antibody (negative control) for one hour. Cells were rinsed in phosphate buffered saline (PBS; 0.15 M NaCl, 8 mM Na_2_HPO_4_, 2.6 mM KCl, 1.5 mM KH_2_PO_4_), and incubated in secondary polymer (Syd Laboratories, Malden, MA), followed by rinsing. Diaminobenzidine reaction was allowed to develop for 5 min, followed by a water rinse and coverslipping. Digital images were captured with a SONY ICX 285AL SPOT camera (Diagnostic Instruments, Sterling Heights, MI). Immunoreactive cells were counted in three groups of 100 per slide, with each stain repeated at least three times. The percentage of cells immunoreactive for each marker was graphed using Prism software (Graphpad, La Jolla, CA). All immunocytochemistry experiments were repeated a minimum of three times and the results combined to generate graphs. Primary antibodies are described in Table 1.

**Table 1:** Primary antibodies used for immunocytochemistry.

Marker	Cell Type	Catalog #, Company, Location	Concentration (ug/ml)
ABCG2	stem cell	ab3380 Abcam, Cambridge, MA	0.5*
Nanog	stem cell	4903 Cell Signaling Technology Danvers, MA	0.094
Oct3/4	stem cell	AP2046c Abgent, SanDiego, CA	2.5
ALDH1A1	stem cell	ab23375 Abcam, Cambridge, MA	1
PAX6	retinal progenitor cell	ab5790 Abcam, Cambridge, MA	3.33
CHX10	retinal progenitor cell	HPA003436 Sigma St. Louis, MO	2.4
RB1	Rb+ cells	9309 (4H1) Cell Signaling Technology, Danvers, MA	N/A

Antibodies were used according to manufacturer's recommendations at the concentrations listed. *=approximate concentration; N/A- not available.

### Reverse transcriptase polymerase chain reaction of stem cell markers

RNA was isolated from frozen cell pellets using TRIzol (Invitrogen) and chloroform (Fisher Scientific, Fairlawn, NJ). Samples were shaken, incubated at room temperature for 2 min and then centrifuged at 12,000 g for 15 min with refrigeration. The aqueous phase was transferred to a fresh tube and mixed with isopropyl alcohol. After 10 min at room temperature, the sample was centrifuged at 12,000 g for 10 min with refrigeration. The RNA pellet was washed twice with 75% ethanol, vortexed, and centrifuged at 7,500 g for 5 min with refrigeration. The pellet was allowed to dry and stored for reverse transcription. Reverse transcription was performed with the iScript cDNA Synthesis kit (Bio-Rad, Hercules, CA), while the PCR reaction was performed using the Promega GoTaq Colorless Master Mix 1-Step PCR (Catalog #M7132, Promega, Madison, WI). Primers used for RT–PCR are listed in Table 2. Fifty microliter samples were generated and 10 µl samples were run in a 2% agarose gel. The PCR protocol was 95 °C for 5 min; 45 cycles with 95 °C for 30 s, 56 °C for 30 s, and 72 °C for 30 s; and 72 °C for 7 min.

**Table 2:** Primers used for RT–PCR.

Gene	Primer sequence (5′ to 3′)	Product size
ALDH1	F: TTACCTGTCCTACTCACCGATT	164 bp
	R: GCCTTGTCAACATCCTCCTTAT	
ABCG2	F: AGCAGGATAAGCCACTCATAGA	341 bp
	R: GTTGGTCGTCAGGAAGAAGAG	
Nanog	F: ATACCTCAGCCTCCAGCAGAT	616 bp
	R: GATTCAGCCAGTGTCCAGACT	
Oct4	F: CCTGTCTCCGTCACCACTC	217 bp
	R: CACCTTCCCTCCAACCAGTT	
GAPDH	F: GAACATCATCCCTGCCTCTACT	184 bp
	R: CGCCTGCTTCACCACCTT	

Primers were obtained from Integrated DNA Technologies, Coralville, IA.

### Quantitative polymerase chain reaction of *RB1*, retinal progenitor markers

mRNA (mRNA) was extracted from cell pellets using the RNeasy Plus Minikit (Qiagen, Valencia, CA) and stored at −80 °C until use. For cDNA synthesis, iScript (BioRad, Hercules, CA) was used for each sample plus “no reverse transcriptase” control. Forward and reverse primers for each marker were obtained from Integrated DNA Technologies (Coralville, IA) and are shown in Table 3. qPCR master mix was assembled using Promega GoTaq colorless master mix (Promega) and SYBR Green dye (Invitrogen). qPCR reactions were run in a MyiQ Cycler (BioRad). Quantitation was based on the equation: 2^-ΔΔCt^, with the glyceraldehyde-3-phosphate dehydrogenase gene (GAPDH) as a reference gene. Graphs were prepared with Prism (Graphpad). All qPCR experiments were repeated a minimum of three times and used different preparations of RB116 cells. Results were combined to generate graphs.

**Table 3:** Primers used for qPCR.

Gene	Primer sequence (5′ to 3′)	Product size
GAPDH	F: TGCACCACCAACTGCTTAGC	87 bp
	R: GGCATGGACTGTGGTCATGAG	
CHX10	F: CTGACTCTGGACCATGCTGA	189 bp
	R: GAGCTGGGAAGGAGGACTCT	
Pax6	F: CACACCGGTTTCCTCCTTCA	80 bp
	R: GGCAGAGCGCTGTAGGTGTT	
RB1	F: GGGTTGTGTCGAAATTGGAT	184 bp
	R: TGTGGCCATTACAACCTCAA	

Primers were obtained from Integrated DNA Technologies, Coralville, IA.

## Results

### Pathology of RB116 tumor

**Figure 1:**
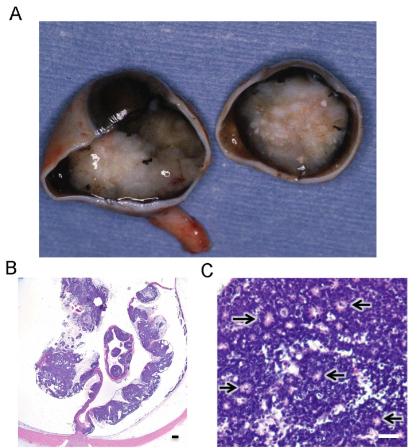
The RB116 tumor is shown with accompanying histology. **A**: The RB116 tumor is shown after enucleation. The tumor fills a significant portion of the globe. **B**: An RB116 tumor section stained with hematoxylin and eosin is magnified 12.5X. The tumor is extensive and displaces the normal retina. **C**: A 400X view of the RB116 tumor reveals Flexner-Wintersteiner rosettes (arrows). The scale bar in panel **B** is one millimeter. The scale bar in panel **C** is 25 microns.

Figure 1A shows the RB116 tumor at the time of enucleation as a large mass filling a significant portion of the globe. Figure 1B,C shows the histopathology of the RB116 tumor at low and high power magnifications. In Figure 1B, it is evident that the tumor displaces most of the normal retina. In Figure 1C, some characteristic Flexner-Wintersteiner rosettes are seen, as indicated by arrows. The RB116 cell line was established from this tumor and analyzed for expression of RB1.

### Expression of *RB1* by RB116 cells

**Figure 2:**
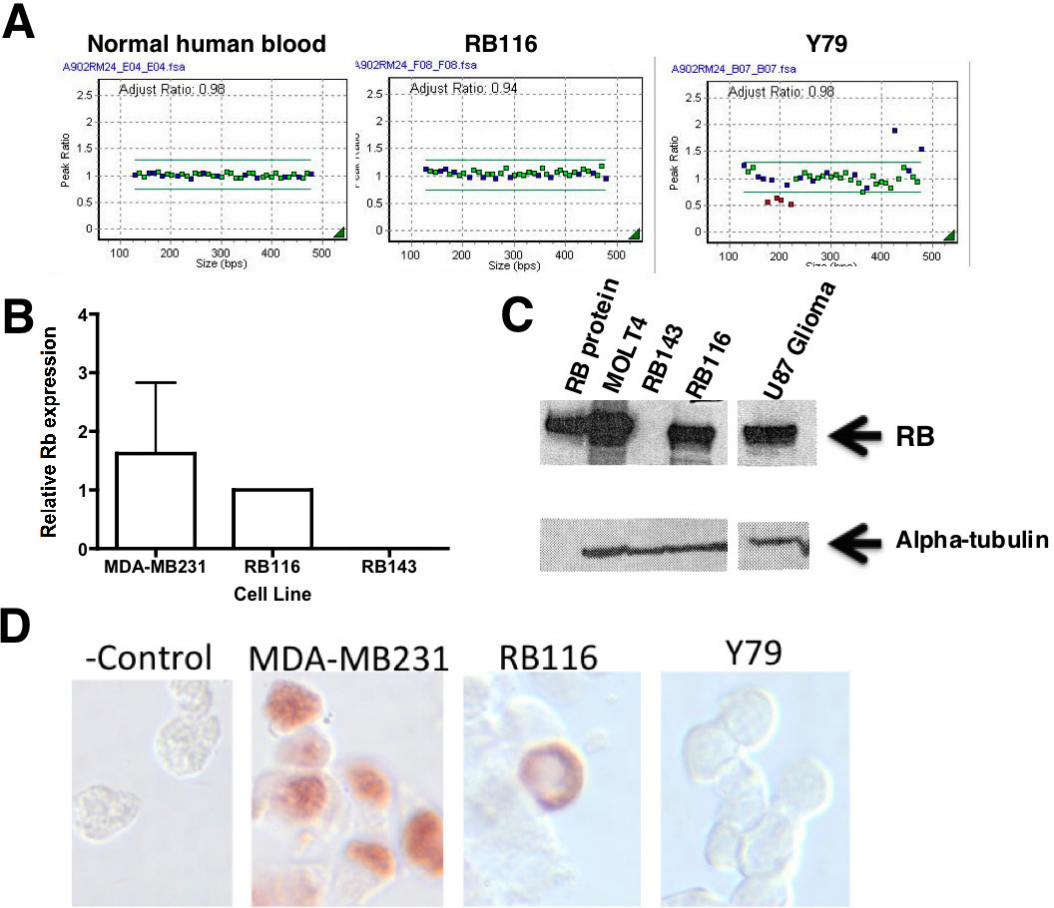
The *RB1* gene and RB1 protein are expressed in RB116 cells. **A**: Multiplex ligation-dependent probe amplification *RB1* (MLPA) is shown for RB116 cells. Gene dosage analysis shows a normal copy number (2) corresponding to all probes, with no duplications or deletions. Probes targeted 23 of RB1’s 27 exons and three nearby genes (*DLEU1, CHC1L, ITM2B*). Y79 cells, with a known multiexon deletion, served as a positive control. Normal human peripheral blood served as a negative control. **B**: Quantitative reverse transcriptase polymerase chain reaction (qRT-PCR) analysis shows *RB1* mRNA expression in RB116 cells. RB116 cells were evaluated alongside RB1-negative RB143 cells and RB1-positive MDA-MB231 breast cancer cells. RB116 cell expression was set at 1.0. The experiment was repeated three times and the error bars show standard deviation. No p values were calculated, as this was an experiment to determine presence or absence of RB1 and not meant to be comparative between cell lines. *RB1* mRNA (mRNA) was detected in both RB116 and MDA-MB231 cells. **C**: western blot analysis detects RB1 protein in RB116 cells. Western immunoblotting was performed on RB116 cells and detected the expected p110 RB1 band that was identical to the positive controls (purified RB1 protein, MOLT4 human leukemia cells, and U87 human glioma cells). No band was seen in the lane containing lysate from the negative control (RB1 negative RB143 cells). The blot was reprobed with alpha-tubulin to ensure equal protein loading of the cell lysates. **D**: Immunocytochemistry demonstrates perinuclear localization of RB1 protein in RB116 cells. RB116 cells were compared with the negative control (Y79 cells) and the positive control (MDA-MB231 cells). The experiment was repeated three times. Note the perinuclear localization of RB1 in RB116 cells. A control slide of RB116 cells received an isotype control antibody instead of the anti-RB1 antibody.

RB116 cells were examined for expression of *RB1* by sequencing, qRT-PCR, western immunoblot, and immunocytochemistry. No variants in the coding region, splice sites, or 5′ proximal promoter region of *RB1* were found by sequencing. In Figure 2, Panel A, MLPA of *RB1* was performed for RB116 cells. Gene dosage analysis showed a normal copy number (2) corresponding to all probes, with no duplications or deletions. Probes targeted 23 of *RB1*’s 27 exons and three nearby genes (*DLEU1*, *CHC1L*, *ITM2B*). Normal human blood and Y79 human RB cells were analyzed for comparison, with the expected results, e.g., the known multiexon deletion in Y79. In Panel B, qRT-PCR analysis was performed to detect *RB1* mRNA in RB116 cells. RB116 cells were compared with RB1-negative RB143 cells and RB1-positive MDA-MB231 breast cancer cells. RB116 cell expression was set at 1.0 for comparison. RB1 was detected in both RB116 and MDA-MB231 cells, but not in RB143 cells. In Figure 2C, western immunoblot analysis was conducted to detect RB1 protein in RB116 cells. A p110 RB1 band was present in the lanes containing: purified RB1 protein, MOLT4 (human leukemia cells), U87 human glioma cells, and RB116 cells. No band was seen in the lane containing lysate from RB1 negative RB143 cells. The blot was reprobed with alpha-tubulin to confirm equal protein loading of the cell lysates. In Panel D, RB116 cells were examined by immunochemical analysis for the cellular localization of RB1 protein as compared with negative and positive controls (Y79 and MDA-MB231 cells, respectively). As expected, RB1 was expressed in the nucleus of MDA-MB231 cells, but not Y79 cells. In addition, there was no staining when RB116 cells were treated with an isotype control antibody. Surprisingly, Rb 116 tumor cells displayed an atypical perinuclear immunoreactivity to RB1.

### Expression of stem cell markers by RB116 cells

**Figure 3:**
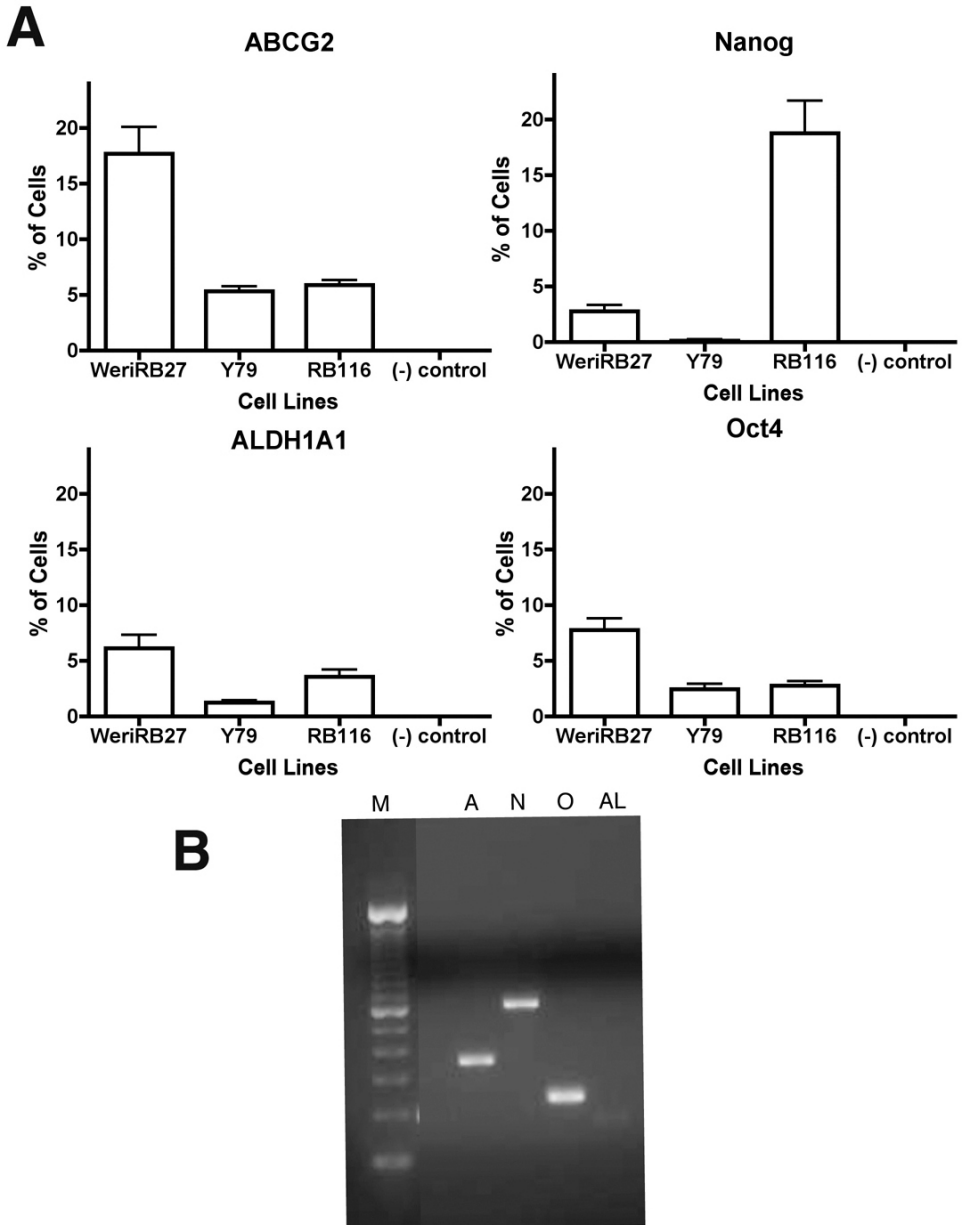
RB116 cells express stem cell markers. **A**: RB116 cells were analyzed by immunocytochemistry, compared with Y79 and WERI-RB27 cells, and found to contain subpopulations of cells that express ABCG2, Nanog, Oct3/4, and ALDH1A1. The (-) control received isotype control antibody instead of primary antibody. Experiments were repeated 3 times and three groups of 100 cells were counted for each cell type. Error bars indicate standard deviation. Tukey’s post tests were performed to calculate p values. For ABCG2, WERI-RB27 is greater than RB116 and Y79 (p<0.001). For Nanog, RB116 is greater than Y79 and WERI-RB27 (p<0.001). For ALDH1A1, WERI-RB27 is greater than Y79 and (-) control (p<0.001) and RB116 is greater than (-) control (p<0.01). For Oct4, WERI-RB27 is greater than Y79, RB116 and (-) control (p<0.001) and RB116 is greater than (-) control (p<0.05). **B**: RB116 cells were analyzed by reverse transcriptase polymerase chain reaction (RT–PCR) and found to express *ABCG2, Nanog, Oct3/4*, and *ALDH1A1*. The lanes of the gel are indicated as follows: M=marker, A=*ABCG2*, n=*Nanog*, O=*Oct3/4*, AL=*ALDH1A1*.

Because our studies had previously demonstrated that stem cell markers are expressed by subpopulations of RB1 negative cell lines, such as Y79 and WERI-RB27 cells (16, 17], we tested RB1+ RB116 cells for the presence of stem cell markers, including ABCG2, Oct3/4, Nanog, and ALDH1A1. As shown in Figure 3A, immunocytochemical analysis revealed immunoreactivity for all four stem cell markers in RB116 cells in comparison with Y79 and WERI-RB27 cells. RB116 cell immunoreactivity was 5.9% for ABCG2, 2.8% for Oct4, 18.7% for Nanog, and 3.6% for ALDH1A1. These same markers were detected in RB116 cells by RT–PCR (Figure 3B).

### Expression of retinal stem cell/progenitor cell markers PAX6 and CHX10

**Figure 4:**
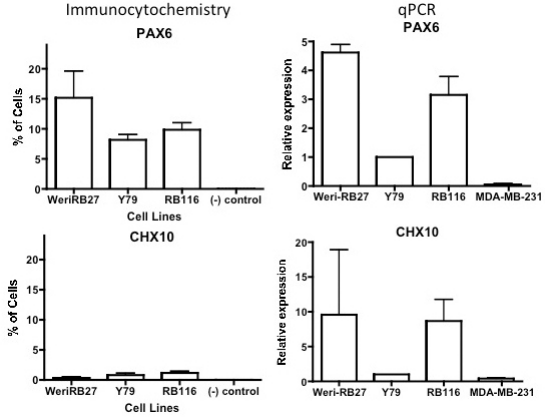
RB116 cells express retinal stem cell/progenitor markers PAX6 and CHX10. RB116 cells were analyzed by immunocytochemistry, compared with Y79 and WERI-RB27 cells, and found to contain subpopulations of cells immunoreactive to PAX6 (9.8%) and CHX10 (1.2%). The (-) control received isotype control antibody instead of primary antibody. Experiments were repeated 3 times and three groups of 100 cells were counted for each cell type. Error bars indicate standard deviation. Tukey’s post tests were performed to calculate p values. For PAX6, WERI-RB27 is greater than (-) control (p<0.001), RB116 is greater than (-) control (p<0.01) and Y79 Is greater than (-)control (p<0.05). For CHX10, one way ANOVA did not reveal differences between cell types at p<0.05. For quantitative PCR (qPCR), Y79 expression was set at 1.0. Both *PAX6* and *CHX10* were detected by qPCR in three experiments. For PAX6, Y79 is greater than (-) control (p<0.05) and RB116 is greater than (-) control (p<0.01). For *CHX10*, one way ANOVA did not reveal differences between the RB cell types at p<0.05. *GAPDH* was used as a reference control gene.

Expression of markers PAX6 and CHX10 was examined in RB116 cells and compared with Y79 and WERI-RB27 cells. (Figure 4). PAX6 was detected in all RB cell lines at both the mRNA and protein levels, with lower levels seen in Y79 cells. For RB116 cells, PAX6 was immunoreactive in 9.8% of RB116 cells. CHX10 exhibited low numbers of immunoreactive cells in all RB cell lines (1.2% in RB116 cells), but did appear to be expressed at higher levels by qPCR in WERI-RB27 cells and RB116 cells.

## Discussion

### Mislocalization of RB1 in RB116 cells

Under normal circumstances, subcellular localization of RB1 is mediated by a nuclear localization signal in the C-terminus of the RB1 protein [21]. The underphosphorylated form of RB1 remains nuclear, in part, due to the association of the N-terminus of RB1 with nuclear matrix proteins [22]. Work by Jiao et al. suggests a role for Cdk phosphorylation-dependent regulation of RB1 subcellular localization [23]. The molecular mechanism(s) underlying RB1 mislocalization in RB 116 cells is beyond the scope of this study; but it is likely due to loss of a nuclear localization function, rather than a lack of RB1 protein itself, as evidenced by strong immunocytochemical reaction of RB1 in RB116 cells and the presence of RB1 protein as seen in the western blot of Figure 2. RB1 mislocalization is not a new phenomenon and has been described as characteristic of more poorly differentiated tumors in a variety of cell types [24]. Therefore, loss of RB1 function via mislocalization may disrupt normal cell differentiation processes and promote tumor progression. These results may explain the development of the original RB116 tumor, despite the lack of mutations in the coding sequence of the *RB1* gene itself.

### Expression of stem cell and progenitor markers in RB116

In previous studies, our group and others have detected stem cell markers in RB1-negative human RB [16,17]. This study represents the first example of RB1+ RB cells expressing stem cell markers, including ABCG2, Nanog, Oct4, and ALDH1A1. These results suggest that mislocalization of RB1/alteration of *RB1* function is compatible with primitive stem cell marker expression, not unlike that seen in RB1-negative cells, such as Y79 and WERI-RB27. Retinal progenitor markers PAX6 and CHX10 are both important in retinal development [25–30], while PAX6 enhances growth and suppresses apoptosis in RB cells [31]. Interestingly, although PAX6 was expressed at both the protein and mRNA levels in all RB cell lines, CHX10 was expressed by 1.2% of RB116 cells, with significant mRNA expression seen by qPCR, suggesting the possibility of a short protein half-life or post-transcriptional regulation of CHX10 protein expression.

Both immunocytochemistry and qPCR confirm that subpopulations of RB116 cells, as well as the RB1-negative cell lines Y79 and WERI-RB27 express primitive markers at both the mRNA and protein levels. We predict that our findings in the RB model system will have broad applications across other tumor models and lead to future studies on the differentiation potential of stem-like cells in RB.
